# Boosting therapeutic efficacy of mesenchymal stem cells in pulmonary fibrosis: The role of genetic modification and preconditioning strategies

**DOI:** 10.22038/IJBMS.2023.69023.15049

**Published:** 2023

**Authors:** Mehrnaz Mehrabani, Sohaib Mohammadyar, Mohammad Amin Rajizadeh, Mohammad Abbas Bejeshk, Bahareh Ahmadi, Mohammad Hadi Nematollahi, Maryamossadat Mirtajaddini Goki, Kobra Bahrampour Juybari, Arian Amirkhosravi

**Affiliations:** 1 Physiology Research Center, Institute of Neuropharmacology, Kerman University of Medical Sciences, Kerman, Iran; 2 Department of Laboratory Hematology and Blood Banking, Faculty of Allied Medicine, Kerman University of Medical Sciences, Kerman, Iran; 3 Department of Physiology and Pharmacology, Afzalipour Medical Faculty, Kerman University of Medical Sciences, Kerman, Iran; 4 Applied Cellular and Molecular Research Center, Kerman University of Medical Sciences, Kerman, Iran; 5 Herbal and Traditional Medicines Research Center, Kerman University of Medical Sciences, Kerman, Iran; 6 Abnormal Uterine Bleeding Research Center, Semnan University of Medical Sciences, Semnan, Iran; 7 School of Pharmacy, Semnan University of Medical Sciences, Semnan, Iran; 8 Pharmaceutical Sciences and Cosmetic Products Research Center, Kerman University of Medical Sciences, Kerman, Iran; 9 Department of Toxicology and Pharmacology, Faculty of Pharmacy, Kerman University of Medical Sciences, Kerman, Iran

**Keywords:** Genetic modification, Preconditioning, Pulmonary fibrosis, Regenerative capacity, Stem cells

## Abstract

Pulmonary fibrosis (PF) is the end stage of severe lung diseases, in which the lung parenchyma is replaced by fibrous scar tissue. The result is a remarkable reduction in pulmonary compliance, which may lead to respiratory failure and even death. Idiopathic pulmonary fibrosis (IPF) is the most prevalent form of PF, with no reasonable etiology. However, some factors are believed to be behind the etiology of PF, including prolonged administration of several medications (e.g., bleomycin and amiodarone), environmental contaminant exposure (e.g., gases, asbestos, and silica), and certain systemic diseases (e.g., systemic lupus erythematosus). Despite significant developments in the diagnostic approach to PF in the last few years, efforts to find more effective treatments remain challenging. With their immunomodulatory, anti-inflammatory, and anti-fibrotic properties, stem cells may provide a promising approach for treating a broad spectrum of fibrotic conditions. However, they may lose their biological functions after long-term *in vitro* culture or exposure to harsh *in vivo* situations. To overcome these limitations, numerous modification techniques, such as genetic modification, preconditioning, and optimization of cultivation methods for stem cell therapy, have been adopted. Herein, we summarize the previous investigations that have been designed to assess the effects of stem cell preconditioning or genetic modification on the regenerative capacity of stem cells in PF.

## Introduction

Pulmonary fibrosis (PF) is the final stage of numerous diffuse diseases of the lung parenchyma. It is described by the accumulation of excessive extracellular matrix (ECM) in parenchymal lung tissue and the formation of fibrotic tissue, resulting in progressive destruction of lung tissue and, finally, high mortality rates of patients (1, 2). 

PF is a life-threatening condition with limited treatment choices. Pharmacological interventions target the inflammatory and fibrotic responses to postpone the progression of PF (3). Thus far, a few antifibrotic medications (pirfenidone and nintedanib) have been approved for the management of idiopathic pulmonary fibrosis (IPF) as the most common form of PF. However, no pharmacological-based treatments have cured PF (4). Over the past few decades, many experimental and clinical studies have investigated the potential of different methods for treating PF (5-10). Approaches based on stem cell therapy are considered innovative and new treatment methods for treating PF (11-15).

Stem cells are undifferentiated cells that can develop into any cell of an organism and can renew themselves (16). In recent years, the therapeutic potential of different types of stem cells, such as induced pluripotent stem cells (iPSCs), embryonic stem cells (ESCs), and adult stem cells (including adipose-derived mesenchymal stem cells, bone marrow mesenchymal stem cells, etc.) have been studied in various investigations (17-22). Despite having extreme pluripotency, including multi-lineage differentiation potential and capability for unlimited self-renewal, therapeutic application of iPSCs and ESCs should be thoroughly considered owing to ethical and safety concerns and low effectiveness (23). In contrast, MSC-based treatment has provided great potential in regenerative medicine due to the self-renewal, multi-lineage differentiation potential, lacking ethical issues, low immunological rejection, immunomodulatory effects, etc. (24, 25). Despite the promising therapeutic potential of MSCs, their native physiological functions have been changed under disease conditions and long-term *in vitro* cultivation (26, 27). Furthermore, when they are systemically administrated and then migrate into the injured tissues/organs, they are confronted with an inhospitable environment associated with inflammation, oxidative stress, and apoptosis (28). 

Thus, a number of approaches such as preconditioning, genetic modification, and optimization of cultivation methods for MSC therapy have been used to overcome the challenges associated with stem cell-based regenerative treatments (29). These strategies have been shown to promote the native physiological functions of MSCs *in vitro* and *in vivo*, thereby resulting in a noticeable improvement in the effectiveness of MSC engraftment in regenerative medicine and tissue engineering (30). The application of these approaches provides the chance to enhance some physiological properties of MSCs such as immunomodulatory, anti-inflammatory, anti-oxidative, anti-apoptotic, and homing properties, as well as accelerates endothelial and epithelial healing (31). Therefore, in the present study, we will summarize preclinical studies that utilize preconditioning or genetic modification strategies to optimize stem cell engraftment in animal models of different pulmonary fibrosis.

**Most recent achievements in the application of MSCs for PF in clinics**

Several studies use MSCs in clinical settings of PF (Table 1). Most clinical trials were conducted in phase 1 and evaluated the safety of MSC therapy in mild to moderated IPF patients. However, to our knowledge, only one study was conducted in phase 2. Averyanov *et al*. aimed to investigate the efficacy of BMSCs in IPF patients with a fast lung function decline. This study was a randomized, placebo-controlled, open-label, phase I/IIA clinical trial. In one group, 1.6 × 10^9^ MSCs were intravenously administrated, and in placebo groups, patients received normal saline. The authors cultured stem cells with autologous serum and platelet lysate instead of FBS. Furthermore, stem cells were maintained in hypoxic (5% О_2_) conditions for the whole duration of culturing. It minimizes oxidative damage to the cell membrane and activates glycolytic processes in stem cells. The results have shown that MSCs therapy increased the lung function of the patients in comparison with the baseline parameters and similar parameters of the placebo group, where a continuous decrease in lung function was detected. The authors claimed that in patients with moderate to severe IPF, high doses of allogeneic MSCs showed logical safety and offered a hopeful method to diminish disease progression (32). 

Another clinical trial was a phase I multicenter clinical trial. 13 patients diagnosed with IPF received an endobronchial dose of autologous adult BM-MSCs. In sum, 46% and 23% of patients did not show functional progression at three months and 12 months, respectively. Stem cell infusion did not induce immediate serious adverse events in patients. However, in severe patients, therapy might be challenging. Genomic instability is another problem the author faced during stem cell therapy (33). 

In another phase 1 clinical trial, nine patients diagnosed with mild to moderate IPF were divided into three groups that received a single IV infusion of 20, 100, or 200 × 10^6^ allogenic human BMSCs. Two patients that received 200 × 10^6^ allogenic human BMSCs died before the completion of a study that did not seem to be related to stem cell therapy. This study has shown the safety of a single infusion of BMSCs in patients with mild to moderate IPF (34).

The other non-randomized, phase Ib clinical trial evaluated the safety of endobronchial administration of pre-activated- autologous adipose-derived stromal cells (ADSCs) -stromal vascular fraction (SVF) at a dose of 1.5×10^6^cells/ kg in patients with mild to moderate IPF. The results have shown the safety of cell therapy and indicated that this method did not worsen patients’ quality of life and functional parameters (35).

Liu *et al*. evaluated the therapeutic effects of MSCs and hepatocyte growth factor (HGF) on human silicosis during a non-randomized uncontrolled trial. PS is an irreversible lung disease characterized by permanent fibrosis in the lungs. Four patients with lung fibrosis received autologous BMSCs modified by a vector containing human HGF cDNA. These cells were intravenously injected at a dose of 2×10^6^ cells/kg weekly for three weeks. The authors have shown the safety of therapy, improved pulmonary function, and a considerable decrease in fibrosis-related symptoms such as chest distress and cough. Furthermore, the serum IgG level and peripheral T lymphocyte subsets were restored to average levels. In sum, the results proposed MSC/HGF administration as a safe and effective treatment for silicosis (36).

A study enrolled 27 COVID-19 patients with PF in their study. The patients received intravenous human embryonic stem cell–derived immunity- and matrix-regulatory cells (hESC-IMRCs) at 3×10^6^ cells/kg. After hESC-IMRC administration, a significant improvement in clinical symptoms was seen. Furthermore, stem cell therapy seemed safe for subjects in the medium term for up to 84 days (37). Another report evaluated the therapeutic safety of placental mesenchymal stem cells (MSC) in eight patients with moderate to severe IPF. The study was a single-center, open-label, non-randomized phase I clinical study. Four patients received a dose of 1×10^6^ placenta-derived MSC/kg, and four patients received a dose of 2×10^6^ placenta-derived MSC/kg. They were followed for six months. It seems both doses were tolerated by patients. However, 25% of them developed small bowel obstruction, and 100% of patients developed halitosis. MSCs infusion was associated with a transient fall in saturation O_2_ after 15 min, while no changes in haemo-dynamics were seen. At six months, the most sensitive markers of disease severity, including Forced vital capacity (FVC) and carbon monoxide diffusion (DLCO), were unchanged compared with baseline, and fibrosis has not worsened (38). Furthermore, without posted data, another study completed phase 1 clinical trial (https://clinicaltrials.gov/ NCT02277145). This study was conducted on the effect of umbilical cord MSCs injection into the lung of patients with radiation-induced PF. The therapeutic safety and efficacy will be measured by a CT density histogram and the patients’ self-evaluation about six months after therapy.

**Genetically modified stem cells for PF**

***Genetic modification***

Despite decades of research and progress in MSC-based tissue repair and renewal, a promising treatment approach against degenerative disorders has yet to be established. Reduced cell viability and imperfect integration of MSCs in the targeted tissue are significant weaknesses in achieving successful therapeutic outcomes in long-term follow-ups (39). Previous investigations in stem cell therapy have indicated that administration of a higher total number of cells even failed to maintain long-term survival and self-renewal of MSCs engraft (40, 41). To overcome these issues, various strategies such as manipulation of the culture conditions, adding medications or cytokines to the MSC culture medium, and genetic modification of MSCs have been recommended to improve the survival rate and therapeutic efficiency of MSCs before infusion (42). Among these, using genetically engineered MSCs is an encouraging approach to expand their therapeutic value *in vivo*. The potential of being genetically manipulated *in vitro* is the most significant feature of MSCs as the cellular vehicle for *in vivo* gene delivery purposes (43). Following transfection with exogenous genes using various vectors, the transgene expression could remain constant without genetic modification even after *in vivo* diﬀerentiation of MSCs. Although genetic manipulation can be generally performed using viral vectors, the usage of non-viral vectors is on the way up (44).

*Application of genetically modified stem cells in preclinical PF models*

Genetic modification of MSCs can enhance the expression of critical factors involved in the therapeutic properties of MSCs (45). Development in the combination of genetic engineering and stem cell research might open up a new and effective way of treating PF (46). Abundant pre-clinical investigations are supporting the effectiveness of using genetically modified MSCs in animal models with PF (Table 2). Survival factors such as bone morphogenetic protein-7 (BMP-7) have been demonstrated to regulate negatively transforming growth factor (TGF)-β1 in renal (47), liver (48), and cardiac fibrosis (49). Augmenting the expression of BMP-7 as an anti-fibrotic cytokine via genetic manipulation of MSCs would be a potential therapeutic approach to preventing lung fibrosis. A recent examination in 2017 indicated that the application of genetically modified MSCs over-expressing BMP-7 could surprisingly alleviate the advancement of silica-induced fibrosis, reducing epithelial-mesenchymal transition in a rat model. Their results revealed that BMP-7 could further enhance the anti-fibrotic therapeutic role of BMSCs. Indeed, the histopathological report demonstrated that the contents of hydroxyproline, fibronectin (FN), and TGF-β1 significantly reduced in the BMP-7-BMSCs-treated group compared to those in the BMSCs group. Furthermore, the authors indicated that the expression of epithelial marker surfactant protein (SP)-C and aquaporin (AQP)-5 significantly increased in the BMP-7-BMSCs-treated group compared to those in the BMSCs group (46).

TGF-β1 is a potent pro-fibrogenic cytokine that prompts the transition of epithelial-to-mesenchymal transition, the apoptosis of epithelial cells, and the formation of other pro-fibrotic mediators, such as VEGF and CTGF (50). Constant release of pro-fibrotic growth factors, such as TGF-β1 by alveolar epithelial cells (AECs), is critical in accelerating the differentiation of fibroblast-to-myofibroblasts in the abnormal healing process of IPF. To treat IPF, rectifying the inadequate connection between epithelial and mesenchymal stroma/stem cells is necessary. MSCs are known to home to the damaged tissues and alleviate lung remodeling by releasing anti-fibrotic humoral mediators in animal models of IPF. Interestingly, in a murine model of bleomycin (BLM)-induced lung fibrosis, MSCs were demonstrated to refine the epithelial-mesenchymal transition through secreting mitochondria-related hormone stanniocalcin-1 (STC1), a survival factor. In this study, researchers transfected the STC1 plasmid to MSCs and augmented its ability to secrete high quantities of STC1 as a reaction to TGF-β1 in response to TGF-β1. Their result showed that STC1 exerted anti-fibrotic effects by diminishing endoplasmic reticulum (ER) and oxidative stress and decreasing TGF-β1 production and collagen-synthesis in the BLM-induced pulmonary fibrosis model (51).

Another signaling pathway connected to fibrosis is angiotensin II (Ag II) signaling. A previous study reported that Ag II could develop fibrosis in a mouse bleomycin-induced pulmonary fibrosis model (52). By contrast, angiotensin-converting enzyme 2 (ACE2) was reported to exert protective effects on severe acute lung injuries induced by acid aspiration, sepsis, and acute respiratory distress syndrome (ARDS) through hydrolysis of Ag II into Ag 1-7 which is in contradiction to the clinical effects of Ag II (53). In line with this report, researchers in 2014 applied ACE2-lentiviral transfection in umbilical cord mesenchymal stem cells (UMSCs) in a mouse model of BLM-induced PF. Their findings recommended that treatment with ACE2-uMSC significantly attenuated lung damage stimulated by bleomycin through a reduction in the expression of oxidation indexes (MDA, oxidized glutathione), fibrosis factors (TNF-α, IFN-γ, and TGF-β), inflammatory cytokines (IL1, IL2, and IL6), tissue inhibitor of metalloproteinase, matrix metalloproteinase (MMP), collagen type 1 mRNA, and hydroxyproline concentration as well as an elevation in the expression levels of IL-10, superoxide dismutase (SOD), ACE2, and glutathione (GSH) (54).

In a different study, uMSCs were transformed using Ad (E1-).decorin to produce decorin-expressing MSCs. Decorin, as one of the natural inhibitors of TGF-β signaling, binds to type I collagen to inhibit the organization of collagen fibrils. In this study, the genetically modified UC-MSCs were intravenously delivered to mice with radiation-induced lung injuries at 6 hr or 28 days after lung irradiation. Finally, histologic lung changes were assessed at 28 days and three months after irradiation. It is noteworthy that uMSCs overexpressing decorin much more effectively stimulated the expression of interferon-γ (IFN-γ), prevented the expression of collagen type III α1 (Col3α1) in injured pulmonary, and reduced the proportion of Tregs in the peripheral blood and spleen. Additionally, DCN-modified MSCs attenuated acute inflammation after radiation therapy and meaningfully prevented later lung fibrosis (55).

Several investigations have proved that MSCs can increase tissue restoration in animal models of pulmonary disease (56-58). MSCs are the source of a variety of growth factors such as EGF, keratinocyte growth factor (KGF), and angiogenic factors (VEGF, PDGF, bFGF, etc), which enhance re-epithelialization and angiogenesis (42, 59-61). Among these, KGF, a member of the FGF family, plays a crucial role in the repair of lung epithelial damage by prompting alveolar epithelial type 2 cell (AEC2) proliferation (62). Given that apoptosis of AEC2 and epithelial destruction are critical events observed in IPF (63), using KGF-modified MSCs could be considered an additional transgenic expression policy to enhance the therapeutic efficacy of the MSCs in PF. However, its clinical efficacy is restricted due to its rapid degradation and inadequate distribution to the distal regions of the lungs without unwanted systemic bioavailability. To achieve a more constant distribution of KGF, Aguilar *et al*. explored the effects of BMSCs and hematopoietic stem cells (HSCs) expressing KGF factor by an inducible lentivirus on PF stimulated by bleomycin in mice. In this study, MSCs and HSCs expressing KGF diminished collagen deposition accompanying BLM-induced PF. They also reported that only HSCs expressing the KGF factor mitigated the histological signs of bleomycin-induced lung fibrosis, stimulated the proliferation of alveolar type II cells (AEC2s), and decreased α-smooth muscle actin (α-SMA) positive myofibroblasts, chemokines, and pro-inflammatory cytokines (64). 

As mentioned above, the genetic modification strategy of MSCs has illustrated promising results in experimental models of IPF (54, 64). However, using genetically modified MSCs for clinical purposes is associated with some challenges that should be highlighted in the future. These challenges can be correlated to the effects of genetic manipulation on the functional characteristics, immunosuppressive properties, and differentiation ability of MSCs. In addition, little good scientific evidence is accessible regarding the prolonged performance of genetically modified stem cells *in vivo*. Furthermore, it is worth paying attention to applying various vectors in modifying MSCs (65). For instance, adverse effects such as chromosomal integration and immunogenicity could be associated with viral transduction, particularly with adenoviral vectors. Therefore, additional exploration is required to consider both the safety and effectiveness of genetically modified MSCs in preclinical models of IPF (42).

***Overexpression or inhibition of specific microRNAs***

*Specific microRNAs*

MicroRNAs (miRs) are an abundant class of small endogenous non-coding RNAs that bind to specific mRNA sequences. MicroRNAs negatively modulate the expression of target genes at the level of mRNAs stability or translation, thereby modulating target protein (s) involved in numerous pathophysiological processes. Besides, the levels of miRs have been found to be up-regulated or down-regulated under different pathological or physiological situations (66). Due to their potential use as a biomarker for early diagnosis, the expression levels of certain miRs have been widely examined in a wide range of specific disorders (67, 68). Furthermore, because the expression of target proteins is intimately related to the abnormal expression of certain miRs, these miRs play a central role in the onset response to pathophysiological stimuli in a disease condition (69).

*MicroRNA function in PF*

Abnormal expression patterns (up-regulation or down-regulation) of miRs have been recognized in the serum of patients with IPF (70) and mouse models of bleomycin-induced PF (71). They interact with several signaling pathways, including wingless-type (Wnt), TGF-β, mothers against decapentaplegic homolog 3 (SMAD3), and β-catenin (63). Limited information about miR function in IPF is available, and most of the information related to miRs has resulted from chemically induced-lung fibrotic murine models. Some examples of *in vivo* studies regarding altered roles of miRs in IPF are summarized as follows (72): (1) miR-21 expression level is significantly elevated in the lungs of mice treated with bleomycin or TGF-β and implicated in the pro-fibrogenic activities of TGF-β through suppressing the transcription of inhibitory Smad7 in the TGF-β/Smad signaling pathway-Smad7, as an inhibitory factor negatively regulates the activity of Smad2 and Smad3 by preventing the binding of Smad2/3 to the TGF-β receptors (69, 73, 74), (2) the expression of miR-155 was also up-regulated in a mouse model of PF. This study recommended that miR-155 possibly modulates fibrosis via regulating Ag II type I receptor (AT1), which is amplified in IPF lungs (75), (3) by contrast, miR-200 family members have been found to prevent the epithelial-mesenchymal transition of AECs induced by TGF-β1. Yang *et al*. showed that miR-200a, miR-200b, and miR-200c were remarkably decreased in the lungs of mice with PF, and restoring miR-200c expression diminished fibrosis (76), (4) Similarly, miR -26a has been shown to be decreased in a mouse model of lung fibrosis. Their results suggested that miR-26a expression level was decreased through TGF-β1-mediated phosphorylation of Smad3, and its over-expression could repress the fibrotic process (77), (5) another study indicated that miR-486-5p over-expression substantially attenuated lung injury severity and its dissemination in mice with BLM- or silica-induced lung fibrosis (78). These findings suggested that miRs as potential molecular targets may propose a novel therapeutic method for modulating PF.

*Application of microRNA-modified stem cells in preclinical PF models*

Given the effectiveness of miRs in animal models of PF, BMSCs have been combined with miRs intended to maximize therapeutic efficacy. In this regard, Huleihel *et al*. demonstrated that BMSCs overexpressing miR-let-7d (anti-fibrotic) or miR-154 (profibrotic) were associated with alterations in the mesenchymal characteristics of BMSCs and their cytokine production in a murine bleomycin model. In this report, bleomycin-injured mice treated with miR-let-7d-modified BMSCs injected intravenously were found to improve earlier than untreated and control MSCs-treated groups. Conversely, mice treated with miR-154-modified BMSCs showed the worst survival. *In vivo* results confirmed that overexpression of miR-let-7d in BMSCs caused a substantial reduction in the gene expression levels of epithelial/mesenchymal markers such as α-SMA, SLUG, N-cad, FSP-1, etc. Let-7d also decreased the collagen content and CD45-positive cells in the injured lung. In addition, weight loss induced by bleomycin was reversed following treatment with miR-let-7d-modified BMSCs. The differential effects of modified stem cells on CD45-positive cells, as a measure of cell inflammation, in the lung possibly are related to their impact on the immunomodulation function. Given that different miRs have been recognized to play significant roles in the pathogenesis of IPF, using several miRs in combination with MSCs may be necessary to make more substantial alterations in PF (79). 

By contrast, Chen *et al*. showed that miR-497-5p was meaningfully elevated during the differentiation of lung resident mesenchymal stem cells (LR-MSCs) into myofibroblast and lung tissues of the BLM-induced PF model. Reversion-inducing cysteine-rich protein with kazal motifs (Reck) was known to be one of the downstream genes of miR-497-5p, and Reck could down-regulate the expression of MMP-2 and MMP-9, which could contribute to the activation of TGF-β1. In this study, the authors managed the expression of miR-497-5p in LR-MSCs and in the lungs of BLM-induced mice to examine the possible therapeutic effects of this miRNA. The results revealed that up-regulation of miR-497-5p could trigger diﬀerentiation of LRMSCs into myofibroblasts and stimulate lung fibrosis in mice. In contrast, suppressing miR-497-5p expression could remarkably repress the abovementioned processes (80). Another research report indicated that suppressing a microRNA named miR-199a-5p restored aged MSCs derived from patients with IPF and recovered their beneficial efficiency in a mouse model of BLM-induced lung fibrosis. Obtained results showed that miR-155-5p prevented autophagy of MSCs via down-regulating the sirtuin 1 (Sirt1)/AMPK signaling pathway, thereby causing cellular senescence. Accordingly, miR-155-5p suppression developed autophagy and improved IPFMSC senescence by triggering the Sirt1/AMPK signaling pathway. In comparison to IPF-MSCs, the administration of anti-miR 199a-5p-IPF-MSCs could retard the progression of PF in mice treated with bleomycin (81).

***Small interfering RNA (siRNA)***

Small interfering RNA (siRNA) refers to a class of double-stranded RNA that contains 21-nucleotide-long guide base pairs in length, comparable with miRNA, and functions in RNA interference. It is also recognized as silencing RNA or short-interfering RNA. It silences the expression of specific genes with complementary nucleotide sequences by breaking mRNA after transcription, inhibiting translation (82, 83).

*siRNA function in PF*

One of the approaches to enhance the therapeutic capability of stem cells in both *in vitro* and *in vivo* experiments involves transporting siRNA into stem cells and influencing the RNAi mechanism (84). Nowadays, siRNA technology, as a natural mechanism to suppress gene expression with superior specificity, has gained special consideration. However, improving a nontoxic, effective, and targetable non-viral delivery of siRNA therapeutics is challenging because of the limited tissue penetration, low circulation consistency, and short circulation lifetime (85). Numerous nanocarriers, such as polyethylene glycol (PEG) and polyethylene imine (PEI), have been designed and used as non-viral gene delivery vectors to overcome these disadvantages. They have sustained blood circulation, excellent tissue/cell permeability, tolerable toxicity, and specific targeting capability (86-88).

*Application of siRNAs-modified stem cells in preclinical PF models*

To demonstrate the abovementioned potential approach for inhibiting PF progression, Ji *et al*. designed micelles generated by a graft copolymer of multiple PEGs modified branched PEI to deliver runt-related transcription factor-1 (RUNX1) siRNA (siRUNX1) to the lung-resident mesenchymal stem cells (LR-MSCs) in the lungs. Accumulating evidence recommends that LR-MSCs play a vital role in the development of PF. RUNX1 has also been shown to be an important regulator of MSC diﬀerentiation. In this research work, the authors found that differentiation of LR-MSCs into myofibroblast was successfully suppressed in a mouse model of BLM-induced PF by administration of siRUNX1-loaded micelles. Moreover, a combination therapy of siRUNX1-loaded micelles and glioma-associated oncogene homolog-1 (Gli1), a vital regulator for myofibroblast diﬀerentiation of MSCs, siRNA (siGli1) caused an outstanding synergistic eﬀect in the treatment of PF and reversed the progression of the disease (89). In addition, researchers showed that Wnt, Fzd, and Gli gene expressions have a critical role in the differentiation of LR-MSCs and the progression of PF. The authors first stimulated the expression of Gli1 in LR-MSCs by TGF-β1 exposure. They indicated that Gli1 enhanced its interaction with the promoter area of Wnt7b/10a to elevate the transcription of these genes. The excessive expression of Wnt7b/10a further induced Wnt/β- catenin signaling through cooperating with their receptor Fzd10. Results obtained from western blotting and q-PCR demonstrated that the suppression of Gli1 prevented myofibroblast differentiation of LRMSCs and PF and alleviated Wnt7b, Wnt10a, and β-catenin expression at protein and mRNA levels. Moreover, transfection of LR-MSCs with LV (lentivirus vector)-Fzd10-siRNA remarkably repressed TGF-β1-stimulated myofibroblast differentiation of LR-MSCs and diminished bleomycin-stimulated PF through the decrease in Fzd10, Col1a1 and α-SMA expression at protein and mRNA levels (90). In line with this report, a research report performed by Shi *et al*. showed that administration of LV-Wnt8b-siRNA could suppress TGF-β1-stimulated myofibroblast differentiation of LR-MSCs *in vitro* and reduce fibrotic lung lesions (91). In the case of siRNA-modified BMSCs, researchers studied the probable impact of silencing non-muscle myosin II (NM-II) on transplanted BMSCs’ survival and therapeutic effects in a rat model of lipopolysaccharide (LPS)-stimulated acute lung injury (ALL). They showed that the transplantation of BMSCs transduced with LV-enhanced green fluorescent protein (eGFP) NMII siRNA effectively promoted histological repair, attenuated the expression of inflammatory cytokines related to ALL, reduced inflammatory infiltration, and decreased interstitial pulmonary edema. Furthermore, NM-II siRNA-modified BMSCs demonstrated antifibrotic characteristics and mitigated the severity of PF stimulated by LPS. In addition, NM-II siRNA-modified BMSCs illustrated somewhat better clinical improvement in pulmonary inflammation when compared to the control BMSCs (92).

**Stem cell preconditioning strategies for pulmonary fibrosis**

**Figure 1:**
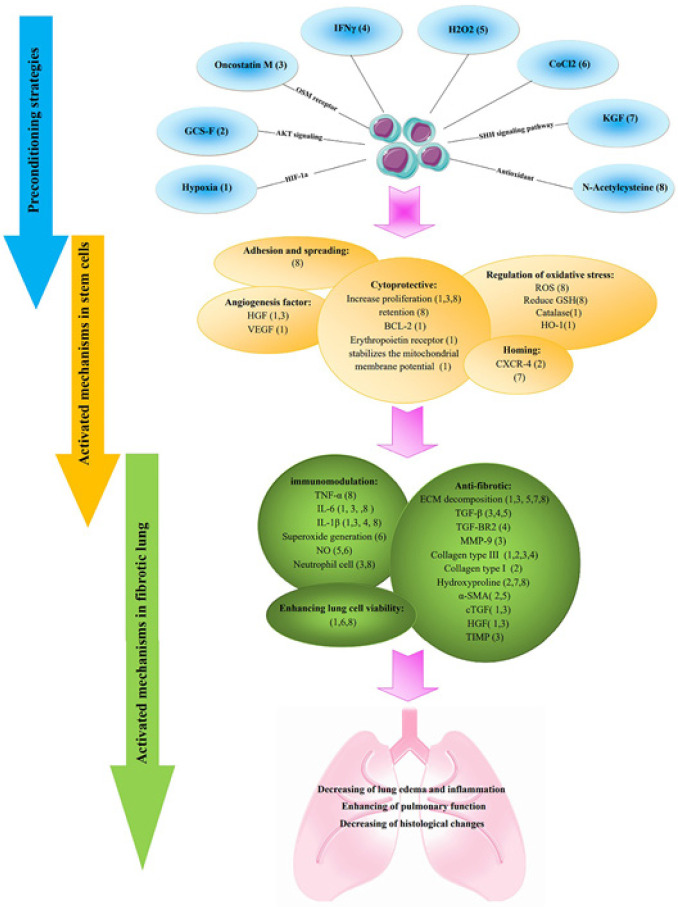
Preconditioning strategies for ameliorating efficiency of stem cell therapy in pulmonary fibrosis (PF)

Preconditioning strategies refer to the approaches that recover stem cells’ therapeutic capacity before transplantation. These approaches have been shown to enhance the survival rate, differentiation capacity, homing ability, and paracrine effect of stem cells (22, 93-96). Some examples of preconditioning methods that have been used are priming with hypoxia, biological molecules and metabolites, pharmacological and chemical agents, physical factors, and serum-free media (31). In this section, we will discuss these preconditioning methods and possible mechanisms that make stem cells more protective against lung fibrosis (Table 3). Various preconditioning methods have been used to enhance stem cell therapy’s efficiency in PF. These approaches target different signaling pathways that are summarized in Figure 1. In the previous research conducted on the different models of lung diseases, including malignant pleural mesothelioma, radiation-induced lung injury, ventilator-induced lung injury, and lipopolysaccharide-induced acute lung injury, different types of methods were performed to increase the therapeutic efficacy of stem cells with promising results (97-101). For example, Li *et al*. used MSCs conditioned by TGF-β1 to increase their survival in lipopolysaccharide (LPS)-induced acute lung injury. They used different doses of TGF-β1 and found that 0.1 ng/ml was the best dose to increase the proliferation of stem cells. Preconditioning by TGF-β1 did not alter the phenotype of MSCs and their differentiation capacity. TGF-β1 was also able to increase ECM expression levels significantly. After using TGF-β1-preconditioned MSCs, the rate of pulmonary edema decreased, and these cells reduced the severity of lung damage (101). In this regard, researchers have used different agents to activate the protective signaling pathways to increase the therapeutic efficiency of stem cells in lung fibrosis.

***Hypoxia preconditioning***

One of these methods is hypoxia priming. Stem cells are usually isolated and cultured under atmospheric oxygen tension (20.9%), which is higher than what they faced in the tissue before transplantation (in the case of MSCs, the oxygen level is about 1.5–4.2%). This process has been shown to negatively affect stem cells’ therapeutic capacity. However, their fates get worse after injection when they are exposed to a deficient oxygen level (0.4–2.3%) in injured tissues. Therefore, one of the significant obstacles in stem cell therapy is the dramatic loss of stem cells after transplantation under lethal hypoxic conditions. Therefore, a re-adjustment strategy to the new hypoxic environment was suggested before cell transplantation, for example, preconditioning MSCs with sub-lethal hypoxia (1–3%) (102). Numerous studies have previously revealed the beneficial role of hypoxic-primed stem cells in different diseases, including acute kidney (103), ischemic conditions such as stroke and myocardial infarction (104-106), traumatic brain injuries (107), diabetes mellitus (108), and liver injuries (109). HP optimized the tissue repairing capacity of stem cells probably by improving their paracrine effect, viability, proliferation, differentiation, and homing capability and also maintaining their stemness besides a reduction in their apoptosis and oxidative stress levels (110). Under hypoxic conditions, VEGF receptor (Flk-1), angiopoietin-1, erythropoietin (EPO), and its cognate receptor (EPOR), Bcl-2, Bcl-xl, and anti-oxidative capacities of cells were enhanced. Also, Caspase 3 activation and cell death were significantly lower in HP-MSC than in normoxic MSC (103, 104, 111). Hypoxia-inducible factors (HIFs) are the most important transcription factors in cellular adaption to hypoxia. HIF consists of two subunits, HIF-α and HIF-β. In the presence of oxygen, 2-oxoglutarate, and iron ions, HIF prolyl hydroxylases hydroxylate the prolyl residues in the oxygen-dependent degradation domain of HIF-1α that are finally recognized by von Hippel-Lindau protein and undergo proteasomal degradation. However, under hypoxic conditions, α subunit was stabilized and dimerized with their β subunits to modulate further the expression of several hundred genes involved in cell proliferation, apoptosis, angiogenesis, glycolysis, etc. (112, 113)*.* So far, three types of HIF-1α, HIF-2α, and HIF-3α, and two types of HIF-1β and HIF-2β have been identified (114). Hypoxia induces a metabolic adaptation in stem cells and increases cellular proliferation, viability, and cytokine secretion in a HIF-1α dependent manner (115). Liu *et al*. researched ischemia/reperfusion (IR) induced lung injury. They cultured mesenchymal stem cells under 1% hypoxic condition. They evaluated the results in four groups: normal, IR, IR + low dose (2.5×10^5^) of hypoxic stem cells, and IR + high-dose (1×10^6^) of hypoxic stem cell groups. A low dose (2.5×10^5^) of hypoxic stem cells exhibited less edema, inflammatory cell infiltration, MPO levels, oxidative parameters, TNF-α, IL-1β, MIP-2 levels, and lung apoptosis. In contrast, the levels of IL-10 and prostaglandin E2 were higher than in the IR group (116). The effect of hypoxia preconditioned stem cells was evaluated by Lan *et al*. in a model of BLM-induced PF. MSCs were exposed to 1.5% oxygen for 24 hr. This study showed that the HIF-1α mRNA level increased significantly 6 hr after induction of hypoxia and reached its maximum level after 12 hr. Also, expression of the multifunctional cryoprotective genes, HIF-1α, HGF, proangiogenic gene, VEGF, and erythropoietin receptor increased. In HP-MSCs, compared to N-MSCs, increased expression of the BCL-2, an anti-apoptotic gene, and anti-oxidant enzymes were observed. They indicated hypoxia stabilizes the mitochondrial membrane potential of stem cells and protects them against H_2_O_2_-induced cell death. The conditioned medium derived for these cells also protects mouse AECs under BLM-induced apoptosis. Hypoxic preconditioned stem cells also attenuated histological changes, inflammation (IL-1β and IL-6), and improved pulmonary respiratory functions in BLM-induced PF in comparison with normal MSCs. The authors noted that the inhibitory effect of HP-MSCs on extracellular matrix production in TGF-β1-stimulated MRC-5 fibroblasts may be driven through HGF (117). The hepatocyte growth factor is activated by hepatocyte growth factor activator (HGFA). Hypoxia-inducible factor-1α has been shown to directly activate the HGFA promoter (118). 

***Biological molecules and metabolites***

Oncostatin M is a 22–28 kD cytokine secreted by leukocytes triggering specific signaling pathways through binding its receptors composed of OSMRβ and gp130 chains. Lan *et al*. employed oncostatin M to enhance the survival of engrafted MSC and their therapeutic efficacy in PF. For this purpose, they treated stem cells with different concentrations of oncostatin M from 0.5 to 5 ng/ml. Oncostatin M at noted concentrations could enhance HGF protein secretion from stem cells. Furthermore, they showed that conditioned medium derived from oncostatin M-preconditioned MSCs (CM-OSM-MSCs) decreased the production of the ECM proteins in TGF-β1 or oncostatin M-stimulated MRC-5 fibroblasts. CM-OSM-MSCs could also exert an anti-inflammatory effect on LPS-treated macrophage and lung tissue by reducing IL-6 and IL-1β. HGF possibly drives these protective effects. In BLM-treated mice, animals showed lower levels of pulmonary edema and infiltration of inflammatory cells in BALF. Besides, the level of tissue inhibitors of metalloproteinase1, TGFβ1, CTGF, collagen type III, and MMP-9 significantly decreased in lung tissues of animals treated with oncostatin M-preconditioned MSCs (OSM-MSCs). Higher pulmonary respiratory functions and lower histological changes were also observed after treating mice with OSM-MSCs compared to MSCs and control groups. Also, OSM-MSCs were more adapted to the microenvironment after administration to the injured lungs (119). 

Researchers studied the therapeutic effects of G-CSF preconditioned MSCs on BLM-induced PF. This study used three doses of MSCs: 1×10^6^, 3×10^6^, and 1×10^7^ cells/ml. PF decreased significantly in 3×10^6^ and 1×10^7^ cells/ml groups, but no improvement in PF was seen in the 1×10^6^ group. However, when MSCs were preconditioned with G-CSF, the rate of fibrosis was significantly lower than that of MSCs. According to these results, it can be understood that the antifibrotic properties of MSCs increase after preconditioning with G-CSF. This study stated that the migration rate of G-CSF preconditioned MSCs to damaged lung tissue was higher than that of non-preconditioned MSCs. G-CSF increases CXCR4 due to activation of the PI3K/AKT signaling pathway, which is why the migration rate is higher in G-CSF preconditioned groups. Also, by knocking down the CXCR4 gene in MSCs, they found that the ability of G-CSF to increase the antifibrotic properties of MSCs was lost (120). In another study, they have shown that G-CSF showed a desirable anti-fibrotic effect in bleomycin-induced PF. For this purpose, G-CSF was injected alone in mice with bleomycin-induced PF at 40 and 60 µg/kg doses, which showed a decrease in fibrosis at dose 40, but no changes were seen at dose 60. They showed that G-CSF can increase the number of stem cells in lung tissue. It was demonstrated that preconditioned bone marrow MSCs using G-CSF (30 ng/ml) showed more homing efficiency than normal MSCs. Their results showed that the expression of CXCR4 in MSCs preconditioned with G-CSF increased both at the mRNA level and the protein level. On the other hand, the level of SDF-1 in BLM-treated lung tissues was significantly increased compared to the control group. As a result, SDF-1/CXCR4 chemotaxis increases the migration of G-CSF preconditioned MSCs to bleomycin-treated lung tissues (121).

 In another study, two types of small-size EVs (ssEVs) and large-size EVs (lsEVs) were extracted from bone marrow-derived MSCs that were preconditioned with INFγ. EVs were then applied to murine with systemic sclerosis to reduce PF. In this study, MSCs were preconditioned by low dose (1 ng/ml) and high dose (20 ng/ml) INFγ. Examination of lung tissue showed that activated EVs with INFγ significantly reduced the expression of fibrotic factors (Col3a1, TGFβ1, and TGFβr2) in the lung tissues compared to inactivated lsEVs. Furthermore, activated lsEVs decreased the production of IL-1β in lung tissue compared to non-activated EVs. Afterward, higher doses of the INFγ (20 ng/ml) were used in preconditioning and showed that EVs secreted from these MSCs had a tremendous potential to decrease α-SMA, TGFβr2 in comparison with inactive EVs. INFγ could modify the expression of several miRNAs in EVs secreted from MSCs. Notably, most miRNA are down-regulated by INFγ. Finally, they showed that several anti-inflammatory factors are increased in INFγ preconditioned MSCs that are not present in the EVs secreted from them (122). IFN-*γ* potentiates the immunosuppressive activity of stem cells by increasing the secretion of the immunosuppressive mediators, including HGF and expression of indoleamine 2,3, dioxygenase that play a vital role in the suppression of T cells (123). INFγ up-regulates B7-H1 as an inhibitory molecule during immune responses and increases the immunosuppression activity of MSCs (124). 

KGF (also known as FGF-7) is considered a key mediator for pulmonary epithelial repair that induces epithelial cell proliferation and differentiation. Since KGF is produced by cells of mesenchymal origin and its role in MSCs and its therapeutic applications has not been elucidated, Yao and colleagues used KGF to pretreat MSCs. KGF-preconditioned MSCs enhanced the protective effect of MSC on the hypoxia-induced PF model through reduction of ECM decomposition and hydroxyproline in lung tissue ultimately leading to decreasing PF. Also, the rate of migration of MSCs to damaged lung tissues increased by KGF. In this study, it was shown that this receptor is strongly expressed in MSCs themselves. It has recently been reported that the sonic hedgehog (Shh) signaling pathway effectively differentiates MSCs. In this study, the effect of KGF on this signaling pathway was measured, and the results showed that KGF could trigger this signaling pathway in MSCs by modulating smoothened, PATCH1, and glioma-associated oncogene homolog 1 (125). 

***Pharmacological and chemical agents***

Wang and colleagues used N-Acetylcysteine for preconditioning MSCs. NAC can eliminate reactive oxygen species (ROS), resulting in more MSC adhesion and spreading. In this study, 25 μM H_2_O_2_ was used to produce ROS products, and these products were severely reduced by treatment with two mM NAC. Also, after treatment, intracellular ROS and reduced glutathione were reduced. In the *in vivo* study, bleomycin was used to induce PF. After treatment with NAC-MSCs, the rate of inflammation and fibrosis in lung cells decreased. Also, in NAC-MSC treatment compared to MSC, the rate of lung cell apoptosis and mortality in mice was reduced (126). H_2_O_2_ is another agent recruited to enhance the therapeutic efficacy of stem cells. In a study by Mahmoudi *et al*., bleomycin was employed to induce PF in mice. Then they were treated with mesenchymal stem cells preconditioned with H_2_O_2_ (15 μmol for 24 hr). This study showed that using H_2_O_2 _preconditioned MSCs can reduce collagen deposition and connective tissue and increase alveolar space. Also, the secretion of pro-fibrotic cytokines such as TGF-β1 and α-SMA, which are highly expressed during the fibrosis process, is reduced by H_2_O_2_ preconditioned MSCs in fibrotic lung tissue. However, there was no significant difference in the activity of the myeloperoxidase enzyme in the two groups treated with MSC and MSC preconditioned with H_2_O_2_ (127). 

***Inefficient preconditioning methods***

Although noted pretreatment strategies enhanced the therapeutic efficiency of stem cells in lung fibrosis, some previous studies showed that some types of preconditioning strategies did not have promising results. For example, in one study, the effect of oxidative stress pre-conditioned MSCs in a mouse model of PF was evaluated. The first result revealed that preconditioning with CoCl_2_ as a hypoxic mimetic agent and H_2_O_2_ did not change the expression of pluripotency marker Oct3/4 and stem cell marker CD105. Besides, it could not alter stem cells’ proliferative ability or induce inflammation in these cells. Furthermore, in a BLM-induced PF model, normal or preconditioned stem cells (hypoxia) could reverse the antiproliferative effect of bleomycin on cells derived from the lungs. Bleomycin could increase superoxide generation in the lung and BALF-derived cells. Normal or hypoxic preconditioned stem cells could decrease it evenly in the lung and BALF-derived cells. However, oxidative preconditioned cells only could reverse the oxidative effect of bleomycin. Only normal cells could decrease BLM-induced NO production in lung cells and all types of cells in BALF cells. Normal or preconditioned stem cells could reverse the histological changes in the lung by reduction of collagen deposition and alveolar thickness after BLM treatment. However, in sum, they concluded that there is no preference for using preconditioned cells compared to non-preconditioned cells (128). Some research also showed that activating a particular signaling pathway may decrease the therapeutic efficacy of stem cells in PF. Peng *et al*. observed that pretreatment of MSCs with NMDA (NmethylDaspartate) could attenuate the therapeutic and anti-fibrotic effects of MSCs on BLM-induced PF. Activating the NMDA receptor inhibits the MAPK/ERK signaling pathway and HGF expression in MSCs. They preconditioned the MSCs with three mM NMDA and then exposed BLM-treated mouse lung epithelial cells (MLE-12) to a conditioned medium derived from NMDA-preconditioned MSCs (MSC-CM). No significant difference in the protein expression level of endoplasmic reticulum (ER) stress markers such as immunoglobulin heavy chainbinding protein, and Xbox binding protein 1 was observed between MSC-CM and BLM groups. However, co-treatment with HGF and NMDA preconditioned MSC-CM increased proliferation and decreased apoptotic rate of MLE-12 cells. This study showed that by activating the NMDA receptor, the protective and therapeutic effects of MSC-CM on BLM-induced PF are inhibited, and these inhibitory effects can be eliminated by using recombinant HGF (129). This group also has shown that treating BM-MSC with NMDA inhibits their migration by blocking the stromal cell-derived factor-1/ CXCR4 signaling axis and decreasing the protective effect of stem cells in lung fibrosis (130). It may also be concluded that preconditioning strategies that manipulated the expression of HGF as an important antifibrotic factor may be considered a promising way to improve stem cells’ therapeutic effect by reducing ER stress and fibrotic and inflammatory markers.

**Current challenges and future perspective**

Stem cell therapy is considered a promising and attractive therapeutic technique, but it includes numerous blind spots and key challenges to clinical application. One of the most profound limitations against translating stem cell preconditioning research into clinical settings is the lack of well-defined and optimal protocols for preconditioning. Preconditioning is an important approach to governing stem cells for clinical applications and developing their efficiency and therapeutic potential. In this regard, further investigations should be performed on innovative cell preconditioning stimuli along with different procedures. In the future, an attitude, which may result in impressive outcomes, is preconditioning with two or more stimuli step by step. In order to enhance the resistance of stem cells, the cells could be first exposed to a potential hypoxia stimulus, and then a non-simulating inducer like a growth factor or an approved medical agent could be applied to prompt cell growth, cell survival, and paracrine capacity of MSCs. Pharmacological preconditioning with anti-inflammatory medications, anti-oxidants, regenerative medicines, etc., could be used to establish and optimize preconditioning. Additionally, obtained results from both *in vitro* and *in vivo* studies illustrated that highly inflammatory and detrimental microenvironments cause premature senescence, thereby, loss of survival signals. In this regard, a combination of modified approaches techniques, critical factors, and medical agents that improve MSC survival, homing capacities, migratory behavior, and adhesion to target sites could further enhance cell survival and expand therapeutic efficiency. However, more in-depth description, characterization, and consequent application of such therapeutic factors/medications could provide future operative clinical benefits for MSC preconditioning. Another crucial limitation is attributed to genetically modified MSCs. It is indicated that the genetic material is generally delivered to the host in a stable integrated manner. However, close management to control random genomic integration in hosts is still necessary. Casual genome integration could result in dangerous genetic dysfunction and potentially malignant mutations. Therefore, the side effects and prolonged influences of the transplantation of genetically modified MSCs should be taken into account. In the future, the underlying mechanisms of gene silencing must be thoroughly investigated to overwhelm the genetic modification of genetically adjusted MSCs shortly after administration. Taken together, some preclinical studies have indicated that modified MSCs can recover lung function, decrease inflammatory cytokines, and repress the progression of PF. However, they are not able to break down the deleterious deposition of ECM, restore the injured alveoli epithelium, or eradicate fibroblasts/myofibroblasts. Therefore, new research studies should be focused on overcoming these impediments associated with the transplantation of modified MSCs.

**Table1:** Application of marrow stem cells for Pulmonary fibrosis in clinics

**Ref**	**Cell type**	**Patient condition**	**Cell delivery and dose**	**Safety**	**Efficacy**
(32)	MSCs cultured under hypoxic conditions with autologous serum and platelet lysate	severe to moderate IPF	Four series (each series included two infusions with 7-day intervals) IV infusions of 200 million cells that was repeated after 12 weeks	No adverse effects reported	After 12 months, remarkable differences were seen in FVC, DLCO, and 6MWDT from the baseline and placebo
(33)	Autologous adult BM-MSCs	mild-to-moderate	In the phase I study, patients divided into three groups received low (10×10^6 ^cells), intermediate (50×10^6^ cells), and high (100×10^6^ cells) doses. In the second phase, patients received the highest tolerated dose	No adverse effects reported	Three patients showed the stability of lung function, and six patients showed functional decline, one patient needed lung transplantation
(34)	Not related, not HLA-matched human bone marrow mesenchymal stem cells to recipients	Mild to moderate IPF	A single intravenous infusion of 20, 100, or 200 x 10^6^	No adverse effects reported	By week 60, a 3.0% mean decline in % predicted FVC, a 5.4% mean decline in % predicted DLCO, and 6-MWT decreased to -4.4% from the baseline
(35)	PRP and photobiostimulation activated autologous adipose-derived stromal cells (ADSCs)-stromal vascular fraction (SVF)	Mild to moderate IPF	Endobronchial infusions of 1.5 million cells per kg of body weight	No adverse effects reported	Did not worsen functional parameters or indicators of quality of life
(36)	Autologous bone marrow MSCs are transfected by a vector containing human HGF cDNA (MSCs/HGF)	Silicosis-related pulmonary fibrosis	IV MSC injections were performed at a dose of 2 x 10^6^ cells/kg weekly for three consecutive weeks	No adverse effects reported	FVC averages were not significantly different before and after treatment. SpO2 was significantly enhanced.
(38)	Placenta-derived stem cells	Mild to not very severe IPF	The patient received an IV infusion of 1 × 10^6^ MSC/kg and 2 × 10^6^ MSC/kg cells	IV MSC administration has a good short-term safety profile	No evidence of worsening fibrosis, so FVC and 6MWD were unchanged after six months, in comparison with the baseline
(37)	Human embryonic stem cell-derived immunity‐ and matrix‐regulatory cells	Mild to severe PF related to COVID‐19	IV transfusion of stem cells at a dose of 3 × 10^6^ cells/kg of body weight once, twice, or thrice	Safe for subjects in the medium term	Some patients can exercise with moderate intensity, and their fibrotic lung lesion areas significantly decreased by 84 days after treatment

FVC: Forced vital capacity; Dlco: Diffusing capacity of the lungs for carbon monoxide; 6MWT: 6-Minute Walk Test; BM-MSCs: Bone marrow stem cells; ADSCs: Adipose-derived stem cells; IV: Intravenous therapy; PRP: Platelet-rich plasma; PF: Pulmonary fibrosis

**Table 2:** Genetically modified stem cells in animal models of pulmonary fibrosis

Ref	Modified genomic target	Stem cell type	Method	Effects on cells or lung tissue	Animal model
**(46)**	BMP-7	BM-MSCs	Overexpression of BMP-7 gene with lentiviral vector	Significant reduction in hydroxyproline, FN, and TGF-β1 contents and a significant increase in the expression of SP-C and AQP-5	Silica-induced pulmonary fibrosis in rats
**(51)**	Stanniocalcin-1 (STC1)	Ue6E7T-2 (BM-MSCs)	Overexpression of STC1 with lentivirus plasmid vector (CMV)	Secretion of STC1 by MSCs diminished the oxidative stress, reduced collagen synthesis, and attenuated ER stress and TGF-β1 production in the BLM-induced pulmonary fibrosis model	BLM-induced pulmonary fibrosis in mice
**(54)**	ACE2	uMSCs	Overexpression of ACE2 with lentiviral vector	Treatment with ACE2‑uMSCs declined the expression of MDA, GSSG, TNF‑α, IFN‑γ, TGF‑β, IL‑1, IL‑2, IL‑6, collagen type 1 mRNA, MMPs, and TIMPs, as well as hydroxyproline concentration, and elevated the expression of SOD, GSH, ACE2, and IL-10	BLM-induced pulmonary fibrosis in mice
**(55)**	Decorin (DCN)	uMSCs	Overexpression of DCN with adenoviral vectors	DCN-expressing MSCs DCN much more effectively stimulated the expression of IFN-γ, prevented the expression of Col3α1 in the injured pulmonary, and reduced the proportion of Tregs in the peripheral blood and spleen	Radiation-induced lung injuries (RILI) in mice
**(64)**	Keratinocyte growth Factor (KGF)	BM-MSCs and Hematopoietic stem cells (HSCs)	Overexpression of KGF with Tet-On inducible lentiviral vector	Both MSCs and HSCs expressing KGF significantly diminished collagen deposition in the injured pulmonary. Only HSCs expressing KGF reduced α-SMA positive myofibroblasts, chemokines, and pro-inflammatory cytokines	BLM-induced pulmonary fibrosis in mice
**(79)**	let-7d	BM-MSCs	Overexpression of let-7d with lentiviral vector	MSCs expressing let-7d resulted in a significant reduction in the gene expression of α-SMA, SLUG, N-cad, etc. It also caused a slight reduction in collagen mRNA levels and decreased the expression of IL-6 levels compared to the other groups. In addition, a distinctive reduction in the rate of CD45-positive cells was observed in let-7d B-MSC-treated mice	BLM-induced pulmonary fibrosis in mice
**(89)**	RUNX1 and Gli1	LR-MSCs	Silencing RUNX1 and Gli1	Successful suppression of myofibroblast diﬀerentiation of LR-MSCs, decrease in the mRNA expression level of RUNX1, Gli1, and α-SMA	BLM-induced pulmonary fibrosis in mice
**(90)**	Fzd10 and Gli1	LR-MSCs	Silencing Fzd10 and Gli1 inhibition	Reduction in the protein expression levels of Wnt7b, Wnt10a, α-SMA, Vimentin, Collagen I, Fzd 9, and β-catenin, preventing TGF-β1-stimulated myofibroblast differentiation of LR-MSCs, and retarding BLM-induced pulmonary fibrosis;	BLM-induced pulmonary fibrosis in mice
**(91)**	Wnt8b	LR-MSCs	siRNA-mediated inhibition of Wnt8b	Reduction in β-catenin, α-SMA, Vimentin, and Collagen I in mRNA and protein levels; Reduction in the mRNA levels of C-myc and cyclin D1 (two Wnt downstream genes related to cell differentiation), preventing TGF-β1-stimulated myofibroblast differentiation of LR-MSCs, and retarding BLM-induced pulmonary fibrosis	BLM-induced pulmonary fibrosis in mice
**(92)**	Non-muscle myosin II (NM-II)	BM-MSCs	siRNA targeted to NM-II mRNA by a lentiviral vector	Attenuating the expression of inflammatory cytokines (IL-1, IL-6, and TNF-α) related to ALL, reducing inflammatory infiltration, and decreasing interstitial pulmonary edema	LPS-induced acute lung injury in rat

BMP-7: Bone morphogenetic protein 7; BM-MSCs: Bone marrow-derived mesenchymal stem cells; FN: fibronectin;TGFB-1: Transforming growth factor beta-1; SP-C: Surfactant protein; AQP-5: Aquaporin-5; STC1: Stanniocalcin-1; BLM: Bleomycin; CMV: Cytomegalovirus; ER: Endoplasmic reticulum; ACE2: Angiotensin-converting enzyme 2; uMSCs: Umbilical cord mesenchymal stem cells; MDA: Malondialdehyde; GSSG: Oxidized glutathione; TNF-α: Tumor necrosis factor alpha; IFN-γ: Interferon gamma; IL: Interleukin; MMPs: Matrix metalloproteinases; TIMPs: Tissue inhibitors of metalloproteinases; SOD: Superoxide dismutase; GSH: Glutathione; DCN: Decorin; Col3α1: Collagen Type III Alpha 1; KGF: Keratinocyte growth Factor; HSCs: Hematopoietic stem cells; α-SMA: Alpha Smooth Muscle Actin; N-cad: N-cadherin; RUNX1: Runt-related transcription factor-1; Gli1: glioma-associated oncogene homolog-1; LR-MSCs: Lung-resident mesenchymal stem cells; Fzd10: Frizzled10; Fzd9: Frizzled 9; Wnt: Wingless-type; siRNA: Small interfering RNA; NM-II: Non-muscle myosin II; LPS: Lipopolysaccharide; ALI: Acute lung injury

**Table 3:** Pharmacological and non-pharmacological preconditioning strategies to promote stem cells potency *in vitro*/*in vivo*

Model	Effects on cells or lung tissue	Affected mechanism in stem cell	Stem cell type	Type of agents	Ref
*In vitro* **: H2O2 treated MSCs, BLM-treated MLE-12 and TGF-B treated MRC-5 cells** **in -vivo: BLM-induced pulmonary fibrosis**	Increase cellular proliferation, attenuation of H2O2-induced cellular cytotoxicity, decrease cell death in BLM-induced apoptosis in mouse alveolar epithelial cells (MLE-12), attenuation of extracellular matrix production, down-regulating the expression of inflammatory and fibrotic factors in lung tissue	HIF-1α, HGF, Erythropoietin receptor, VEGF	BM-MSCs	Hypoxia (1.5%)	(117)
*In vitro* **:** **TGF-B-stimulated MRC-5 fibroblast, LPS-stimulated macrophage** **;** ***In*** *-vivo* **: bleomycin-induced pulmonary fibrosis**	Increase proliferation of stem cell and migration of MRC‐5, decrease total collagen content and fibroblasts in TGF-B-stimulated MRC-5, decrease pro-IL-1β and IL-6 in LPS-stimulated macrophages, anti-fibrotic, anti-inflammatory in lung tissue	overexpress type 2 OSM receptor (gp130/OSMRb) and HGF	BMMSCs	Oncostatin M (OSM) Pre-treatment (2 ng/ml 24 hr)	(119)
**bleomycin-induced pulmonary fibrosis**	Decrease protein levels of α-SMA and collagen content in lung tissue by promoting BMSC homing to injured lung tissues	Akt / CXCR4	BMMSCs	Granulocyte colony-stimulating factor (G-CSF) pre-treatment (30 ng/ml)	(120)
*In vitro* **: TGF-b stimulated fibroblast, ** *in vivo* **: bleomycin-induced pulmonary fibrosis**	Attenuating the proliferation and trans-differentiation of FBs, promoting the homing ability of MSCs by the CXCR4/SDF-1 axis	CXCR4	BMMSCs	Granulocyte colony-stimulating factor (G-CSF) pre-treatment 30 ng/ml)	(121)
**Murine with Systemic sclerosis (SSc)**	decreaseی TGF-B COL3a1 TGFβr2 IL-1β and improved the antifibrotic and anti-inflammatory effects of MSC	miRNAs	MSCs	IFNγ Pre-treatment low dose (1 ng/ml) and high dose (20 ng/ml)	(122)
**Hyperoxia-induced lung fibrosis model**	Alleviation of ECM, hydroxyproline deposition, and increased mobilization of stem cell	SHH pathway (SMO, PATCH1, and GLI1)	BM-MSCs	KGF (10 ng/ml)	(125)
*In vitro* **: H2O2 exposed MSCs (25 mmol/L)** *In vivo* **: bleomycin-induced injured lungs**	Decrease inflammation and fibrosis, collagen deposition, hydroxyproline content, cellular infiltration, and inflammatory cytokines	Decrease ROS production, increase GSH level, increase spreading and adhesion of stem cells	Human embryonic MSCs	N-Acetylcysteine (2 mmol/L)	(126)
*In-vivo* **: bleomycin-induced pulmonary fibrosis**	Decrease TGF-β1 and α-SMA in the parenchyma areas of lung tissues, anti-fibrotic, reduce collagen deposition and connective tissue, and increase alveolar space	__	Human umbilical cord vein-derived MSCs (hUCV-MSCs)	H2O2 sub-lethal concentration (15 μM for 24 h)	(127)
**bleomycin-induced pulmonary fibrosis**	preconditioning did not significantly improve the beneficial effect of MSC administration	__	BM-MSCs	CoCl2 (100 µM) H2O2	(128)

BM-MSCs; Bone marrow mesenchymal stem cells; HIF- 1α; Hypoxia-inducible factor 1-alpha; HGF; Hepatocyte growth factor; VEGF; Vascular endothelial growth factor; BLM; Bleomycin; MSCs; Mesenchymal stem cells; TGF-β; Transforming growth factor beta; LPS; Lipopolysaccharide; CXCR4; C-X-C motif chemokine receptor 4; BMSC; Bone marrow mesenchymal stem cells; α-SMA; Smooth muscle alpha-actin (αSMA); FBs; Fibroblasts; SDF-1; Stromal cell-derived factor-1; SHH; Sonic Hedgehog Signaling; SMO; Smoothened; PATCH1; Patched-1; GLI1; Glioma-Associated Oncogene Homolog 1; ECM; Extracellular matrix; ROS; Reactive oxygen species; GSH; Glutathione

The number refers to a specific priming method

## Conclusion

It is becoming clear that PF constitutes a challenge for basic and clinical investigators. Accumulation of data in preclinical studies suggests that stem cell-based therapy plays a significant role in lung repair after injury. However, the harsh microenvironment of the injured tissues/organs, low cell survival, and engraftment of transplanted MSCs severely hamper the therapeutic effects of MSCs. Latest developments in the field of regenerative medicine, such as cell preconditioning and genetic modifications, promoted the therapeutic potential of transplanted MSCs via different strategies. However, there are still many complications that should be considered to standardize the optimal techniques for preconditioning and genetic modification in stem cell-based therapy. Firstly, genetically engineered MSCs may be associated with safety concerns and lead to unpredictable therapeutic consequences. Secondly, preclinical studies evaluate the efficacy of manipulated MSCs in only lung fibrotic models, while most of the patients with lung fibrosis develop an infection, cardiovascular disease, lung cancer, and other health problems or are old. Thirdly, animal models do not entirely reflect all processes that take place in humans, and the correlation between the results of preclinical models and conducting clinical trials should be thoroughly considered. Hence, it may be possible that these procedures are not influential enough to battle such a situation. Accordingly, more focal investigations are required to overcome disadvantages and determine the optimal strategies.

## Authors’ Contributions

M M, S M, A A, MA R, MA B, B A, MH N, and M MG contributed to the writing of the manuscript. KBJ was involved in writing and planning and supervised the work. MM also designed the figure and tables.

## Funding

This research received a grant from the Physiology Research Center, Institute of Neuropharmacology, Kerman University of Medical Sciences, Kerman, Iran (grant number: 401000485). This study was approved by the Ethics Committee of Kerman University of Medical Sciences (IR.KMU.REC.1401.454).

## Conflicts of Interest

The authors declare no conflicts of interest.
